# Evolutionary Patterns in the Sequence and Structure of Transfer RNA: A Window into Early Translation and the Genetic Code

**DOI:** 10.1371/journal.pone.0002799

**Published:** 2008-07-30

**Authors:** Feng-Jie Sun, Gustavo Caetano-Anollés

**Affiliations:** Department of Crop Sciences, University of Illinois at Urbana-Champaign, Urbana, Illinois, United States of America; University of Cape Town, South Africa

## Abstract

Transfer RNA (tRNA) molecules play vital roles during protein synthesis. Their acceptor arms are aminoacylated with specific amino acid residues while their anticodons delimit codon specificity. The history of these two functions has been generally linked in evolutionary studies of the genetic code. However, these functions could have been differentially recruited as evolutionary signatures were left embedded in tRNA molecules. Here we built phylogenies derived from the sequence and structure of tRNA, we forced taxa into monophyletic groups using constraint analyses, tested competing evolutionary hypotheses, and generated timelines of amino acid charging and codon discovery. Charging of Sec, Tyr, Ser and Leu appeared ancient, while specificities related to Asn, Met, and Arg were derived. The timelines also uncovered an early role of the second and then first codon bases, identified codons for Ala and Pro as the most ancient, and revealed important evolutionary take-overs related to the loss of the long variable arm in tRNA. The lack of correlation between ancestries of amino acid charging and encoding indicated that the separate discoveries of these functions reflected independent histories of recruitment. These histories were probably curbed by co-options and important take-overs during early diversification of the living world.

## Introduction

Modern day proteins are synthesized in ribosomes, complex molecular machines made of proteins and RNA. The relatively small L-shaped tRNA adaptors are central to protein biosynthesis and establish numerous interactions with important macromolecules in addition to ribosomal RNA [1]. Specific amino acids are charged to the acceptor arms through the activity of cognate aminoacyl-tRNA synthetases (aaRSs), while the ‘anticodon’ arms contain triplets of bases that recognize complementary ‘codon’ sequences in messenger RNA. These interactions shape the genetic code, delimit the identity, degeneracy, and function of tRNA, and are therefore fundamental to our understanding of how the biosynthetic machinery and the genetic code were set up into place in an emergent world of proteins and organisms.

It seems commonly accepted that early in the history of life only few amino acids were encoded and that most of the possible codons were fairly soon brought into use [2]. However, the composition of the initial group of amino acids that was used by ancient translation systems has been controversial. Numerous groups of amino acids have been suggested as candidates ([3] and reference therein) and many hypotheses have been proposed to explain the underlying genetic code. For example, the well-known co-evolution theory postulates that the expansion of amino acids is achieved by biosynthetic transformation of precursor amino acids into product amino acids [4]. According to this hypothesis, the earliest encoded proteins were made up of pre-biotically synthesized amino acids, specifically Gly, Ala, Ser, Asp, and Glu. Three phases of amino acid entry into proteins were later proposed, in which amino acids originated first from pre-biotic synthesis (Gly, Ala, Ser, Asp, Glu, Val, Leu, Ile, and possibly Pro and Thr), later from protein-mediated biosynthetic pathways (Arg, His, Met, Trp, Asn, Gln, Lys, and possibly Phe, Tyr, and Cys), and then from post-translational modification without direct genetic encoding [5]. While the co-evolution theory is popularly supported [6]–[8], it has been criticized and remains controversial [9], [10]. For example, a group of four amino acids (Gly, Ala, Asp, and Glu) were proposed to be the first to enter the biosphere [11]. This group was later redefined by replacing Asp and Glu with Arg and Pro and postulating that families of related amino acids evolved from the initial amino acids, as the genetic code expanded [12], [13]. Yarus [14] suggested that Arg was the first amino acid, based on the unique nature of its RNA binding site. In a synthesis effort, Trifonov [3] used 60 criteria to propose a chronology of appearance of amino acids and their respective codons, each of which provided a temporal order. The order of amino acid appearance followed the sequence Gly, Ala, Asp, Val, Pro, Ser, Glu, (Leu and Thr), Arg, (Ile, Gln, and Asn), His, Lys, Cys, Phe, Tyr, Met, and Trp, with the earliest 10 amino acids (from Gly to Arg) being synthesized in the imitation experiments of Miller [15], [16]. However, this boundary may be unrealistic because Miller [16] also indicated that with the possible exception of only three amino acids (Arg, Lys, and His), all other amino acids could be derived from pre-biotic synthesis. Amino acid usage rates have also been used to infer evolution of amino acids and the genetic code, using either asymmetries in substitution matrices among closely related organisms [17], [18] or ancestral sequence reconstructions of ancient protein lineages [19], [20]. Brooks et al. [19] showed that nine amino acids (Ala, Asn, Asp, Gly, His, Ile, Ser, Thr, and Val) had a decreased frequency in proteins and could have been introduced early into the genetic code, once organismal diversification was in place. However, Jordan et al. [18] recently revealed a quite different group of early amino acids with declining frequencies in proteins (Ala, Glu, Gly, and Pro). These probabilistic methods and their implicit assumptions were recently questioned [21], and a more stringent approach of only counting fully conserved positions in ribosomal proteins was used to propose that Gln, Gly, Leu, and possibly Pro, Asp, and Asn were encoded earlier, while Cys, Phe, Glu, Ile, Val, Trp, Tyr, and possibly Lys, Glu, and Ser were late additions to the genetic code. It is quite clear that our understanding of how tRNA function has evolved is far from complete.

In this study we use information embedded in the sequence and structure of tRNA molecules to study the history of amino acid charging and encoding. We first build an intrinsically rooted global tree of tRNA molecules using a well-established cladistic method [22], [23] that embeds RNA structure directly into phylogenetic analysis [24]. The approach was previously used to study evolutionary patterns in ribosomal RNA, spacer RNA, short interspersed element RNA, and many other functional RNA molecules [22], [23], [25]–[27][Sun and Caetano-Anollés, unpublished], and in particular, the origin and evolution of the major structural and functional components of tRNA [28]. Since tRNA embeds a history of recruitment in which structures gain or co-opt new identities and functions or take over established ones in processes that restrict the acquisition of phenotypic traits or functions in lineages, we sorted out these confounding processes by forcing monophyletic groupings of taxa (sets that share a common ancestor) during tree building to test alterative hypotheses or establish evolutionary timelines of structural and functional diversification [27]. This phylogenetic method (known as constraint analysis) is powerful and was recently used to gain insight into the origin of cellular superkingdoms and viruses [27]. Here, the method opens an unanticipated window into early translation and the genetic code.

## Results

**Figure 1:**
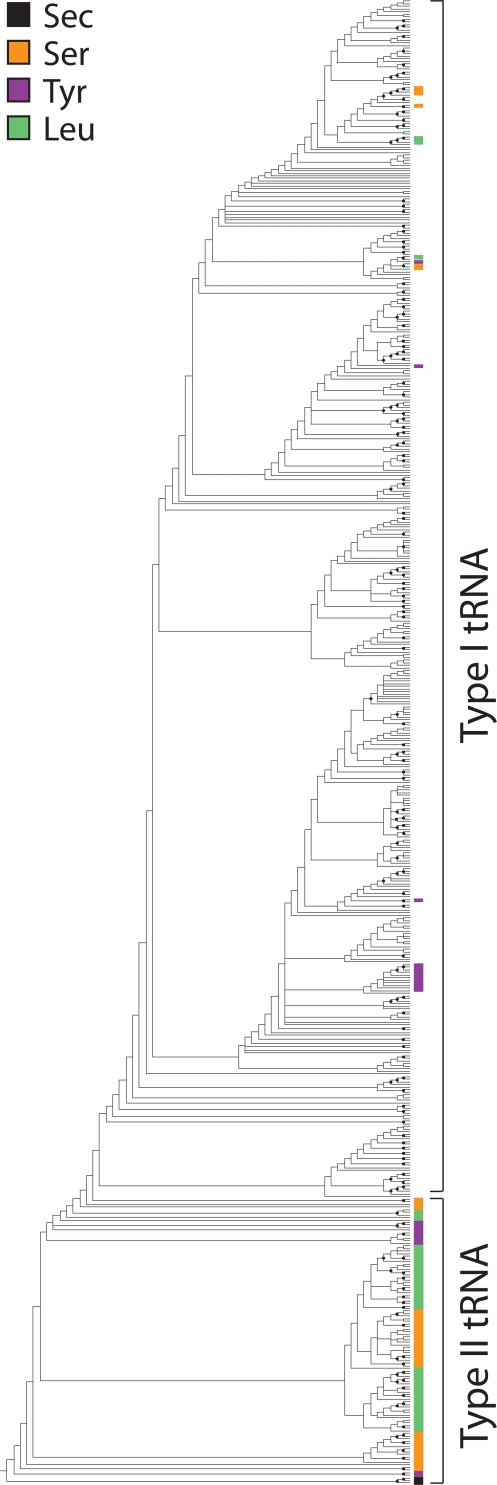
A global phylogenetic tree of tRNA molecules reconstructed from sequence and structure. MP analyses of data from 571 tRNA molecules resulted in the preset limit of 20,000 minimal length trees, each of 10,083 steps. Consistency index = 0.069 and 0.069, with and without uninformative characters, respectively; Retention index = 0.681; Rescaled consistency index = 0.047; g_1_ = −0.107. Terminal leaves are not labeled since they would not be legible. tRNA molecules coding for Sec, Ser, Tyr, and Leu are labeled with colors. Note several of these tRNAs have short variable arms and these are derived in the tree. Nodes labeled with closed circles have BS values >50%. The figure has been modified from [27] and a global tRNA tree with labeled terminal taxa can be found in Supporting Information (Figure S1).

We generated rooted universal trees of tRNA from the sequence and structure of 571 tRNAs representing part 2 of the bayreuth trna database. This data set contains information on modified bases, molecules from organisms in the three superkingdoms of life and viruses, and all isoacceptor variants and amino acid specificities [27]. The optimal most parsimonious trees had lengths of 10,083 steps and were intrinsically rooted (Figures 1, S1, and S2). Bootstrap support (BS) values were generally low for most clades, an expected observation given the large number of taxa (molecules) analyzed. In fact, there were 497 branches that defined more than one taxon, 180 of which were supported by BS>50% and generally defined 2–7 taxa, except for only one composed of 14 tRNAs with specificity for Phe. In other words, the branches at levels closer to terminal taxa were supported with relatively higher BS values. Specifically, 24, 23, 29, 21, and 83 branches out of the 497 defining the tree were supported by 50–60%, 61–70%, 71–80%, 81–90%, and 91–100% BS values, respectively. Most of the 83 branches that were highly supported defined only 2–3 taxa (only 6 defined 4–7).

**Figure 2:**
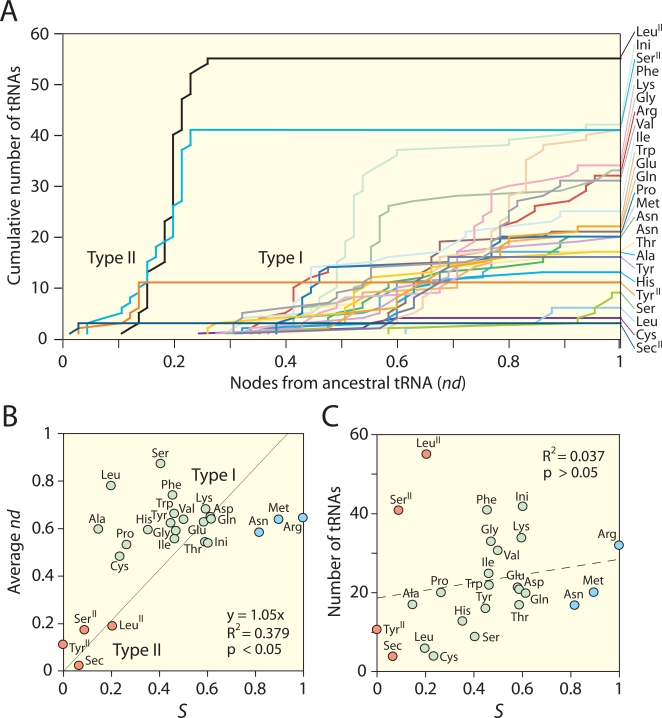
Phylogenetic analyses of the amino acid charging function in tRNA. A. Cumulative frequency distribution plots that describe the cumulative number of tRNAs charging for different amino acids as a function of node distance (*nd*), the number of nodes from an hypothetical tRNA molecules at the base of the tree. B. Average *nd* for each tRNA charging group plotted against number of additional steps (*S*) needed to satisfy constraints that force the monophyletic grouping of corresponding tRNA molecules, normalized to a 0–1 scale. Different colored circles correspond to the three groups of tRNA molecules described in the text. C. Plot describing the effect of numbers of tRNA that are constrained versus normalized *S* values.

Type II tRNA molecules with long variable arms, including tRNA^Sec^ and most tRNA^Ser^, tRNA^Tyr^, and tRNA^Leu^ isoacceptors, appeared at the base of the rooted trees as a paraphyletic group (Figure 1). Similarly, trees reconstructed from 17 data matrices partitioned according to organismal identity that had more than 10 taxa and contained type II tRNAs, always placed type II tRNA molecules at their base (Figure S3, Table S1). To unfold the data embedded in the trees, we plotted cumulative number of tRNA isoacceptors expressing different amino acid specificities as a function of distance in nodes from a hypothetical ancestral tRNA molecule (node distance, *nd*) [23] in the trees (Figure 2A). These plots showed clearly the basal placement of type II tRNA molecules, but trees failed to reveal groupings that would indicate clear evolutionary links to organismal origin or molecular functions. The monophyly of tRNAs belonging to each superkingdom (or viruses) or expressing different amino acid specificities was not revealed. Similarly, tRNAs with specificities for previously proposed ancestral amino acids [3], [16], [18], [19], [21] or sharing the first, second, third, or the first two bases in codons did not form monophyletic groups. These patterns were also observed in trees derived from partitioned matrices of superkingdoms or viruses (data not shown).

In order to uncover evolutionary patterns and test alternative hypotheses we forced groups of tRNAs related by functions (amino acid charging specificity and codon identity) into monophyly using constraint analyses. We then examined the length of the most parsimonious trees that were obtained and the number of additional steps (*S*) that were needed to force the constraint. This exercise was generally done either with or without forcing types I and II tRNA molecules into separate groups, but overall results were congruent. The values of *S* for constraints related to amino acid charging specificity ranged from 113 steps for tRNA^Sec^ to 255 for tRNA^Arg^ or from 130 for tRNA^Tyr^ to 266 for tRNA^Arg^, with or without forcing types I and II tRNA molecules into groups, respectively (Table 1). These values delimited the following consensus chronology of amino acid charging, starting with the most ancient charging functions and ending with the most recent: (Sec^II^, Tyr^II^), (Ser^II^, Leu^II^, Leu^I^, Ala^I^, Cys^I^, Pro^I^), His^I^, Ser^I^, (Tyr^I^, Phe^I^, Ile^I^, Trp^I^), Gly^I^, (Val^I^, Glu^I^), (Thr^I^, Lys^I^, Ini^I^, Asp^I^), Gln^I^, Asn^I^, Met^I^, and Arg^I^ (subscripts indicate tRNA types and parentheses indicate groups of functions that cannot be dissected in the timeline). Lower *S* values corresponded to ancient tRNAs in the timeline and this trend was derived from the rooted trees (and embedded assumptions of polarization; see Materials and Methods). These tendencies were for example confirmed when ancestries of isoacceptor groups derived from cumulative frequency distribution plots (expressed as average or minimum *nd* values) were plotted against *S,* normalized to a 0–1 scale (Figure 2B). Three tRNA groups were evident: type II tRNA isoacceptors which were basal in the trees and could be constrained by few additional steps (low *S*), type I tRNA isoacceptors that were more derived and had larger *S*, and a derived group of three type I tRNAs (tRNA^Asn^, tRNA^Met^, and tRNA^Arg^) with the largest *S* values. In these analyses, the number of tRNAs in a group did not affect *S*. For example, *S* values for isoacceptor groups were not correlated to the number of tRNA molecules that were constrained (Figure 2C). This is expected since molecules analyzed generally exceed by far those that were constrained.

**Table 1:** The numbers of additional steps (*S*) required to force molecules into monophyly based on constraints related to amino acid specificity (*spec*) or amino acid specificity and tRNA structural classes (type I and II tRNAs) (*cat-spec*) during MP analyses of the combined structure and sequence data for 571 tRNA molecules.

Constraint: *spec*	*S_spec_*	Constraints: *cat-spec*	*S_cat-spec_*
((Tyr^II^), …)	133	((Type I: Ala, Arg, …, Val), (Type II: (Tyr), Sec, Ser, Leu))	130
((Sec), …)	113	((Type I: Ala, Arg, …, Val), (Type II: (Sec), Ser, Leu, Tyr))	139
((Ser^II^), …)	140	((Type I: Ala, Arg, …, Val), (Type II: (Ser), Sec, Leu, Tyr))	142
((Leu^II^), …)	146	((Type I: Ala, Arg, …, Val), (Type II: (Leu), Sec, Ser, Tyr))	158
((Ala), …)	148	((Type I: (Ala), Arg, …, Val), (Type II: Sec, Ser, Leu, Tyr))	150
((Leu), …)	140	((Type I: (Leu), Ala, …, Val), (Type II: Sec, Ser, Leu, Tyr))	157
((Cys), …)	151	((Type I: (Cys), Ala, …, Val), (Type II: Sec, Ser, Leu, Tyr))	162
((Pro), …)	144	((Type I: (Pro), Ala, …, Val), (Type II: Sec, Ser, Leu, Tyr))	166
((His), …)	161	((Type I: (His), Ala, …, Val), (Type II: Sec, Ser, Leu, Tyr))	178
((Ser), …)	166	((Type I: (Ser), Ala, …, Val), (Type II: Sec, Ser, Leu, Tyr))	185
((Tyr), …)	173	((Type I: (Tyr), Ala, …, Val), (Type II: Sec, Ser, Leu, Tyr))	191
((Phe), …)	179	((Type I: (Phe), Ala, …, Val), (Type II: Sec, Ser, Leu, Tyr))	192
((Ile), …)	167	((Type I: (Ile), Ala, …, Val), (Type II: Sec, Ser, Leu, Tyr))	193
((Trp), …)	175	((Type I: (Trp), Ala, …, Val), (Type II: Sec, Ser, Leu, Tyr))	193
((Gly), …)	186	((Type I: (Gly), Ala, …, Val), (Type II: Sec, Ser, Leu, Tyr))	194
((Val), …)	188	((Type I: (Val), Ala, …, Tyr), (Type II: Sec, Ser, Leu, Tyr))	198
((Glu), …)	187	((Type I: (Glu), Ala, …, Val), (Type II: Sec, Ser, Leu, Tyr))	210
((Thr), …)	203	((Type I: (Thr), Ala, …, Val), (Type II: Sec, Ser, Leu, Tyr))	210
((Lys), …)	199	((Type I: (Lys), Ala, …, Val), (Type II: Sec, Ser, Leu, Tyr))	211
((Ini), …)	190	((Type I: (Ini), Ala, …, Val), (Type II: Sec, Ser, Leu, Tyr))	212
((Asp), …)	194	((Type I: (Asp), Ala, …, Val), (Type II: Sec, Ser, Leu, Tyr))	213
((Gln), …)	207	((Type I: (Gln), Ala, …, Val), (Type II: Sec, Ser, Leu, Tyr))	214
((Asn), …)	231	((Type I: (Asn), Ala, …, Val), (Type II: Sec, Ser, Leu, Tyr))	241
((Met), …)	235	((Type I: (Met), Ala, …, Val), (Type II: Sec, Ser, Leu, Tyr))	252
((Arg), …)	255	((Type I: (Arg), Ala, …, Val), (Type II: Sec, Ser, Leu, Tyr))	266

Each constrained group is given in parentheses and amino acids are indicated by the IUPAC 3-letter nomenclature. The length of the most parsimonious trees derived from the combined data set was 10,083 steps. Superscripts associated with amino acid codes indicate tRNAs belong to Type II molecules.

Constraining tRNA molecules based on amino acids synthesized in Miller's experiments [16] was more parsimonious (*S* = 339) than ancient tRNA groups circumscribed by Trifonov [3], Brooks et al. [19], Jordan et al. [18], or Fournier and Gogarten [21] (*S* = 357–566; Table 2). Remarkably, only 135–253 steps were needed to force type II tRNA molecules containing the long variable arms into monophyly.

**Table 2:** The numbers of additional steps (*S*) required to force molecules into monophyly based on amino acid chronology constraints during MP analyses of combined tRNA structure and sequence data.

Chronology	Constraints	*S*
Miller [16]	((Gly, Ala, Asp, Val, Pro, Ser, Glu, Thr, Leu, Ile), …)	339
	((Gly, Ala, Asp, Val, Pro, Ser, Glu, Thr, Leu, Ile), (…))	357
Brooks et al. [19]	((Cys, Trp, Tyr, Gln, Phe, Leu, Lys, Glu), (Ala, Val, Gly, Ile, Thr, Asp, Ser, Asn, His), …)	516
	((Cys, Trp, Tyr, Gln, Phe, Leu, Lys, Glu), (Ala, Val, Gly, Ile, Thr, Asp, Ser, Asn, His), (…))	530
Trifonov [3]	((His, Lys, Cys, Phe, Tyr, Met, Ini, Trp, Sec), …)	332
	((His, Lys, Cys, Phe, Tyr, Met, Ini, Trp, Sec), (…))	348
Jordan et al. [18]	((Cys, Met, Ini, His, Ser, Phe, Asn, Thr, Ile, Val), (Pro, Ala, Glu, Gly, Lys), …)	550
	((Cys, Met, Ini, His, Ser, Phe, Asn, Thr, Ile, Val), (Pro, Ala, Glu, Gly, Lys), (…))	566
Fournier and Gogarten [21]	((Cys, Glu, Phe, Ile, Lys, Val, Trp, Tyr, Ser), (Asn, Gln, Gly, Leu, Pro, Asp), …)	524
	((Cys, Glu, Phe, Ile, Lys, Val, Trp, Tyr, Ser), (Asn, Gln, Gly, Leu, Pro, Asp), (…))	546
Present study (Type I and II are combined)	((Ser, Sec, Leu, Tyr), …)	139
	((Ser, Sec, Leu, Tyr), (…))	253
Present study (Type I and II are separated)	((Ser, Sec, Leu, Tyr), …)	135
	((Ser, Sec, Leu, Tyr), (…))	232

The length of the most parsimonious trees derived from the combined data set was 10,083 steps. Each constrained group is given in parentheses and groups of tRNA isoacceptors were labeled with IUPAC 3-letter amino acid nomenclature.

**Figure 3:**
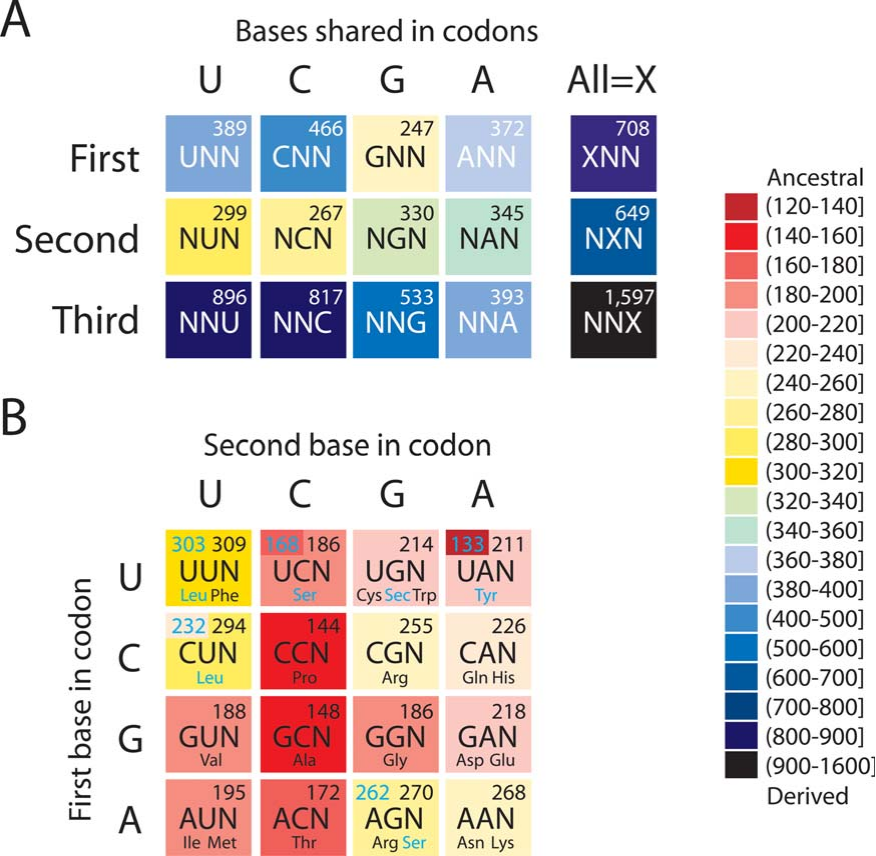
Phylogenetic analysis of codon identity functions in tRNA. A. Degenerate genetic code table painted with colors describing the ancestry (*S*) of their codon identity function, in which two (A) or one (B) position in the codon is degenerate and harbors any one of the four bases (N). Ancestries were derived by constraining sets of tRNA molecules into monophyletic groups. *S* values are provided in the right hand corner for every codon, and corrected *S* values that exclude type I molecules from the constraints are given in the left hand corner for codons related to Leu, Ser, and Tyr. Amino acid that are encoded are listed below corresponding codons.

When constraining tRNAs according to codon identity (Table 3), we found that forcing tRNAs sharing the second bases in codons (*S* = 267–345) was more parsimonious than forcing tRNAs sharing the first (*S* = 247–466) or third bases (*S* = 393–896) in codons into monophyly (Figure 3A). The same trend was evident when all groups of tRNA sharing bases in different codon positions were constrained (Figure 3A). Constraining molecules sharing first and second bases in codons showed that tRNAs sharing C in the second base of codons required less steps (*S* = 144–186) than tRNAs sharing G (*S* = 186–270), U (*S* = 188–309), or A (*S* = 211–268), in that order (Figure 3B). With one exception (CGN coding for Arg), all ancestral placements involved codons with strong/moderate base interactions (5–6 hydrogen bonds for the first two positions). Codons with weak base interactions were generally associated with larger *S*.

**Table 3:** The numbers of additional steps (*S*) required to force molecules sharing the first, second, third, or the first two bases in codons into monophyly during MP analyses of the combined tRNA structure and sequence data.

Constraints	*S*
Shared the first bases in codons:	
((ANN), …)	372
((CNN), …)	466
((GNN), …)	247
((UNN), …)	389
((ANN), (CNN), (GNN), (UNN))	708
Shared the second bases in codons:	
((NAN), …)	345
((NCN), …)	267
((NGN), …)	330
((NUN), …)	299
((NAN), (NCN), (NGN), (NUN))	649
Shared the third bases in codons:	
((NNA), …)	393
((NNC), …)	817
((NNG), …)	533
((NNU), …)	896
((NNA), (NNC), (NNG), (NNU))	1597
Shared the first and the second bases in codons:	
((AAN), …)	268
((ACN), …)	172
((AGN), …)	270
((AUN), …)	195
((CAN), …)	226
((CCN), …)	144
((CGN), …)	255
((CUN), …)	294
((GAN), …)	218
((GCN), …)	148
((GGN), …)	186
((GUN), …)	188
((UAN), …)	211
((UCN), …)	186
((UGN), …)	214
((UUN), …)	309
((AAN), (ACN), (AGN), (AUN), (CAN), (CCN), (CGN), (CUN), (GAN), (GCN), (GGN), (GUN), (UAN), (UCN), (UGN), (UUN))	1197

The length of the most parsimonious trees derived from the combined data set was 10,083 steps. Each constrained group is given in parentheses and groups of tRNA molecules are indicated by codons with shared the first, second, third, or the first two bases. Symbol “N” indicates A, U, G, or C.

**Figure 4:**
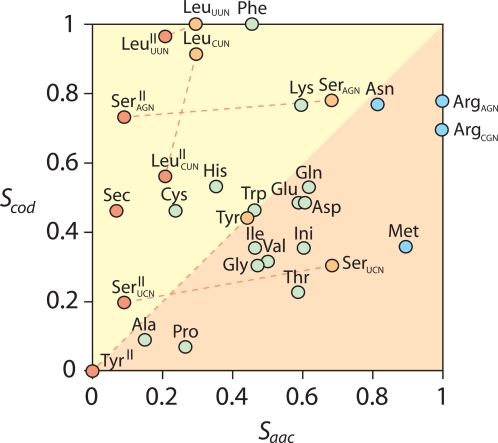
Plot describing the relationship between ancestries of amino acid charging (*S_aac_*) and codon identity (*S_cod_*) function, normalized to a 0–1 scale. Dashed lines describe the effect of excluding type I tRNA variants from the constraints (from orange to red circles), and illustrate recruitment events related to the loss of the variable arm in these molecules. Color schemes of circles follow those of Figure 2.

A plot of amino acid charging ancestries (*S_aac_* for amino acid charging constraints) versus codon ancestries (*S_cod_* for codon constraints) showed poor correlation (*p*>0.05) between timelines of amino acid charging and codon discovery (Figure 4). In particular, ancestral Sec, Tyr, Ser, and Leu charging functions had codons that were derived. Exclusion of type I tRNA from codon constraints for these amino acids resulted in the recovery of the basal placement of the Tyr coding function, but not of the rest. These results underscore the evolutionary significance of separate recruitment processes involving amino acid charging and codon discovery.

## Discussion

### Deep evolutionary patterns embedded in tRNA phylogenies

In order to uncover evolutionary patterns related to amino acid charging and the genetic code, we first generated rooted phylogenetic trees using information in the sequence and structure of tRNA (Figures 1 and S1). We chose part 2 of the bayreuth trna database because it contains detailed information on base modifications known to be important determinants of tRNA structure and because it represents the most complete dataset at RNA level currently available. However, we note that tRNA molecules in part 2 are unbalanced in term of representation of organisms in superkingdoms (e.g., overrepresentation of Haloarchaea in archaeal tRNAs, or *Saccharomyces* in eukaryal tRNAs) or in isoacceptor composition (e.g., underrepresentation of tRNA^Cys^). We also note that other tRNA databases, including part 1 of the bayreuth trna database, include genomic compilations of tRNAs identified in organisms that were generally selected based on technical (e.g., model systems), medical (e.g., pathogenic or obligate parasitic microbes), or biotechnological (e.g., crops of importance) criteria. These other databases are also biased and affect our ability to sample appropriately the living world for patterns embedded in tRNA molecules.

As shown previously [27], [28][Sun and Caetano-Anollés, unpublished], type II tRNA molecules with long variable arms coding for Sec, Ser, Tyr, and Leu appeared at the base of the rooted trees and were ancient. However, we were unable to reveal any other patterns of significance in the trees. In particular, there were no clear monophyletic groupings related to molecules with similar amino acid charging functions or codon identities (e.g., Figure 2A). The basal placement of type II tRNAs and the absence of clear monophyletic groupings were also observed in trees reconstructed from partitioned data matrices (Figure S3) and from a preliminary study of 1,029 tRNA sequence and structures from part 1 of the bayreuth trna database (genomic tRNA compilation at DNA level) [Ospina, Sun and Caetano-Anollés, unpublished]. This larger set has a more balanced distribution of organismal origins and isoacceptor identities but lacks information on base modifications. Overall results indicate that the basal placement of type II tRNAs is robust and relatively independent of database biases in tRNA representation.

In order to unravel the intricate history of tRNA, we explored competing (alternative) or non-competing phylogenetic hypotheses by reconstructing sub-optimal trees containing constrained monophyletic groupings of taxa [27]. Competing hypotheses were quantitatively contrasted based on the number of additional steps (*S*) relative to the optimal tree, and those that were more parsimonious were not rejected. We used this approach to test for example competing hypotheses of amino acid chronology. In contrast, non-competing hypotheses were ranked by the values of *S* and were used to define timelines of amino acid specificities and codon discovery. Hypotheses with smaller values of *S* (more parsimonious) were considered less affected by the confounding effects of recruitment in lineages and represented processes that were more ancient. In other words, lineages defined by these hypotheses merged (coalesced) in backwards time more easily to fit the constraint. We have validated this fundamental assumption of ‘polarization’ by mapping the correlation between *S* and number of nodes from a hypothetical tRNA ancestor in the trees (Figure 2B). This type of approach is known as ‘constraint analysis,’ a procedure commonly used in cybernetics to deconstruct systems into their component parts [29] or in phylogenetics to test hypotheses of monophyly [30].

The analysis is supported by two fundamental assumptions. First, we assume tRNA structures recruited new identities and functions as the genetic coded expanded, and that different structures were co-opted in different lineages and different functional contexts. Recruitment is pervasive in evolution of macromolecules and has been demonstrated in cellular metabolism, where protein enzymes are often recruited from one pathway to another to perform new enzymatic tasks [31]–[33]. At RNA level, tRNA structural diversification appears to have predated organismal diversification [27], [34][Sun and Caetano-Anollés, unpublished] and the functions and identities affiliated to present-day tRNA structures probably evolved in lineages and were swapped by horizontal gene transfer events in evolution. Second, we assume old tRNA structures developed or recruited new functions (co-options) more often than new tRNA structures acquired old functions (take-overs), an assumption that is supported by global studies of enzyme recruitment in metabolism [Kim et al., unpublished]. The trees show several instances of take-overs, indicating modern type I structures lacking the variable arms took over ancient amino acid charging functions associated with type II structures (Figure 1)[27][Sun and Caetano-Anollés, unpublished]. As they spread through lineages, old structures have more chances to succeed in a diversifying world while younger structures are restricted to the lineage in which they originated, and can only spread further through horizontal gene transfer events. In other words, older functions associated with tRNA will be less prone to co-options than younger functions. Consequently, ancient molecules sharing functions or belonging to selected lineages will be more easily constrained than younger variants in phylogenetic reconstruction. This clearly unfolds in Figure 2B.

The analysis also depends on the validity of our evolutionary models and associated assumptions of character polarization. Phylogenetic reconstruction produces trees that are rooted according to specific models of character transformation, i.e. models that define how individual phylogenetic characters transform from one character state to another along the branches of the trees. In contrast with standard phylogenetic methods, our models include a central hypothesis or axiom that invokes an evolutionary search of conformational order in molecules which defines the general direction of the evolutionary path [22], [23], [25]–[28]. Trees are therefore rooted without the need and associated uncertainties of local external hypotheses of relationship (e.g., the use of ‘outgroup’ taxa). We note however that the validity of the models that we use is well-supported by statistical mechanic, thermodynamic, and phylogenetic considerations. Character argumentation is described in detail in Materials and Methods.

Any phylogenetic analysis rests on how strongly the data support the topology of the tree, and our tRNA phylogenies are no exception. Tree reconstruction showed the existence of well-resolved tRNA relationships but revealed low consistency indices (CI) and BS values (Figure 1). However, this should not be construed as statements of poor reliability, especially because of the large number of taxa that are present in our global tRNA tree. Based on previous predictions and observations, Sanderson and Donoghue [35] confirmed an inverse relationship between taxa and CI. This results from the increase in the number of cladogenic events that is expected when taxa are added to a tree, which also increases the chances of homoplasy. Note however that character change is also significantly and non-trivially correlated with CI and could explain more variation in homoplasy than taxa (i.e., CI values reflect more than conflict in phylogenetic reconstruction)[36]. In fact, we used simulation data from [36] to extrapolate CI levels (∼0.6) that are present in 56 partitioned tRNA datasets with ∼15 taxa [28](e.g., Table S1) to levels in the global data matrix with 571 taxa (assuming ∼5 character changes per branch [27]) and found that the CI decreased to ∼5×10^−5^, which is significantly lower that the value we observe (6.9×10^−2^). Similarly, bootstrap and jackknife measures of topological stability (nodal reliability) are also inversely correlated with number of taxa, and their usefulness in assessing support for branches is therefore artifactually limited in large-scale (>100 taxa) phylogeny reconstructions [37]. As additional taxa are added to a tree, the information that supports each branch is diluted and at the same time the support for overall relationships is enhanced [37]. This limitation is especially severe in cases of small character number, nicely illustrated in a careful comparison of molecular and morphological characters in Rubiaceae [38]. Moreover, the effects of sampling on BS values are not only dependent on taxon number but also on the search algorithm [39]. Considering that 27% of branches in our global tRNA tree, at 0.25 characters per taxon, had BS values >75%, the reliability of our tree compares well with phylogenies describing the evolution of 163 *rbc*L sequences from the Rubiaceae, at 5.19 characters per taxon, that have been used as standard in a study of effects of taxa and characters on tree reliability [38]. Since addition of characters to an analysis has a substantial positive effect on reliability [37], the performance of our dataset vastly exceeds that of the *rbc*L dataset and should be construed as acceptable, given the low character-taxon ratios utilized. To conclude, while many object philosophically to bootstrapping (mainly because of lack of proper specification of an underlying probability distribution) and its usefulness with large-scale phylogenies remains debated, nodal support levels obtained in this study compare well (if not exceed) those in the recent literature. A cursory exploration of robustness of published large-scale phylogenies (including our own phylogenomic global tree reconstructions [33]) reveal BS levels comparable to those in the *rbc*L phylogeny that we have chosen here as reference. Finally, we also assume phylogenies are free from systematic errors and the confounding effects of mutational saturation, long branch attraction, and unequal rates of evolution along branches of the tree. We do not address these issues in this paper since they have been discussed in [27].

Using the same strategy we apply here, we recently established an evolutionary timeline of organismal diversification [27]. The study showed that the lineage of Archaea segregated from an ancient community of ancestral organisms early in evolution. We also demonstrated that organismal diversification predates the discovery of modern amino acid charging. A separate line of evidence also supports this conclusion [34]. Here, we focus on timelines of amino acid charging and codon discovery.

### Timelines of amino acid charging specificity

We constrained each and every group of tRNA molecules coding for specific amino acids and ranked them according to the values of *S* (Table 1). This ranking defined a timeline for the amino acid charging function (see Results) that separated ancient type II molecules coding for Sec, Tyr, Ser, and Leu from the rest of tRNAs, and placed type I molecules coding for Asn, Met, and Arg as the most derived group (Figure 2B). The most ancient type I tRNA molecules in the timeline are those coding for Leu, Ala, Cys, and Pro. These tendencies matched patterns in the trees that were revealed by cumulative frequency plots (Figure 2A). It is noteworthy that the amino acid group charged by ancient type II tRNAs includes Ser and Leu, which have 6 codons each, the most for one amino acid in the table of the genetic code, and are among the most abundant amino acids that can be synthesized in a variety of pre-biotic environments [16]. Our timeline also suggests Arg was added very late in evolution, rejecting the proposal of it being the most ancestral amino acid [14]. Our results are incompatible with chronologies that have been previously proposed. Table 2 shows that results derived from the sequence and structure of tRNA are incompatible with ancient tRNA groups defined earlier [3], [18], [19], [21]. In particular, lack of congruence among groups defined by Trifonov [3], Brooks et al. [19], and Jordan et al. [18] indicates that the relative use of amino acids in modern biochemistry is a feature that may not be directly related to tRNA function, which is mostly delimited by the biochemistry of identity elements in the acceptor, anticodon, or variable arms of the tRNA molecules [40].

### The early origin of the Sec charging function

Our timeline clearly supports the ancestral nature of the Sec charging function. Sec, one of the two non-canonical amino acid residues (the other is Pyl), is introduced into proteins during translation under the direction of UGA, a typically stop codon which also codes for Cys [41] and Trp [42]–[45]. Uniquely, Sec is synthesized co-translationally on tRNA^Sec^ [46], [47] without a cognate aminoacyl-tRNA synthetase and the tRNA^Sec^ (designated as tRNA^[Ser]Sec^) is initially aminoacylated with Ser [48]–[53]. Seryl-tRNA synthetase (SerRS) forms Ser-tRNA^Sec^ which is conversed into selenocysteyl-tRNA^Sec^ in all three domains of life, Bacteria [51], Archaea [54], [55], and Eukarya [56]. In Bacteria, the formation of Sec from Ser is achieved in a single step by Sec synthase. In both Eukarya and Archaea, an additional phosphorylation step is required, catalyzed by *O*-phosphoseryl-tRNA^Sec^ kinase (PSTK) and converting the resulting *O*-phosphoeryl-tRNA^Sec^ (Sep-tRNA^Sec^) to Sec-tRNA^Sec^ by Sep-tRNA:Sec-tRNA synthase (SepSecS) [57], [58]. Phylogenetic analyses have shown that PSTK co-evolved precisely with SepSecS and that the archaeal and eukaryotic PSTKs originated before the evolutionary divergence of the superkingdoms Archaea and Eukarya [59].

The origin of Sec has remained uncertain and controversial [60]. Two strikingly opposing hypotheses have been proposed to explain its evolutionary ancestry. On one hand, it was suggested that UGA was originally a sense codon for Sec, one of the earliest amino acids to be charged, and later evolved into a new coding function, such as termination or Trp codons in the case of mycoplasma or mitochondria [51], [61]. The use of Sec could have been counter selected by the introduction of oxygen into the earth's atmosphere. This excluded the use of this highly oxidizable amino acid except in anaerobic or well-protected chemical environments. This scenario was supported by the discovery of proteins with high contents of Sec in a symbiotic δ-proteobacterium of a gutless worm [62]. However, it was also suggested that anaerobic environments could actually support the use of Sec [62], [63]. On the other hand, it was argued that Sec evolved in the later stages of the development of the genetic code [64].

The Sec moiety is part of the active center [49], [65] in most enzymes that contain Sec [61]. Three hallmarks characterize the Sec utilization system: (i) Sec is always encoded by UGA, (ii) the incorporation of Sec always requires a stem-loop specificity sequence—the SECIS element, and (iii) there is always a dedicated translation elongation factor plus an RNA-binding component. These hallmarks support the concept of a common ancestor. Phylogenetic analysis demonstrated that bacterial, archaeal and eukaryotic selenocysteine incorporation machineries already existed at the time of the last universal common ancestor [57]. This also strongly supports the hypothesis that all life began with the opportunity to utilize Sec, and that Sec utilization has been lost by many groups of organisms during evolution, most likely due to the limited supply of selenium [66]. This is consistent with the observation that organisms have only a limited number of selenoproteins and that so many organisms lack selenoproteins altogether. Together with our observations, these results strongly support the ancestral nature of Sec and the co-translational insertion of Sec in the genetic code prior to the separation of the three superkindoms of life. It also agrees with the early evolutionary history of SepSecS, the enzyme that catalyzes the formation of Sec-tRNA^Sec^, that shows tRNA-dependent Sec formation is a primordial process [67].

### Timelines of codon discovery and the evolutionary significance of the second codon position

The standard genetic code maps a set of 64 (4^3^) base triplets (codons) to 20 standard amino acid molecules, plus Sec [61] and Pyl [68] for organismal subsets, and 3 translation stop signals [69]. The code has a non-random design, in which similar amino acids are generally delimited by codons that differ in the first and second positions [2]. Therefore, it is highly redundant. When we constrained tRNA molecules sharing the first, second, or third bases in codons to form monophyletic groups, molecules sharing the second bases had the lowest *S* values (Figure 3A). Their codons should be considered ancestral. Molecules sharing the first position in codons had higher *S*, and those sharing the third position had the highest *S* values. Their codons were clearly more derived. This result suggests the early code was molded by the second and then the first codon position, a conclusion consistent with a large body of evidence. Constraint analyses also showed that when forcing both the second and first bases of a codon, *S* values were minimal when the second base was C, particularly when the number of hydrogen bonds established by the first two base pairs was maximal (6 hydrogen bonds; codons CCN and GCN) (Figure 3B). The values of *S* were also low when the first base was G. These results suggest that early codes involved CCN and GCN motifs and that later these codes expanded to include ACN and then UCN, GGN, GUN, and AUN motifs. Interestingly, CGN motifs encoding for Arg were introduced late in evolution, consistent with the very late charging of this amino acid. Consequently, codon-anticodon directionality was very important during origins of the genetic code and primordial double strand coding [70]–[72]. Our timeline suggests the first codon groups to appear belonged to CCN and GCN, an observation that supports the proposal that the very first codons originated from the GCU triplet and its point change derivatives [73]. The late appearance of codons for Sec and Tyr by codon capture is also supported by related work [3].

It has been argued that similar codons correspond to similar amino acids because the earliest forms of translation were imprecise, and the distant ancestors of tRNAs were only able to encode classes of similar codons (an extreme form of wobble) and classes of similar amino acids [74]–[76]. These classes of similar amino acids could have shared the same chemical or biological properties. As far as similar amino acids are concerned, Woese et al. [75] found that U in the second position codes for amino acids with hydrophobic side chains and that amino acids coded for by C in the second position seem to have consistently similar polar requirement. This observation was further supported by a multivariate study of the relationship between the genetic code and the physical-chemical properties of amino acids [77]. A relationship existed between the physical-chemical properties of the amino acids and which of the A, U, or C nucleotide was used in the second codon position. However, the amino acids coded for by G in the second codon position did not participate in this relationship. Haig and Hurst [78] calculated the average effect of changing a codon by a single base for all possible single-base changes in the genetic code and for changes in the first, second, or third codon positions separately. They concluded that amino acids whose codons differed by a single base in the first and third codon positions were very similar with respect to polar requirement and hydropathy, and that the major differences between amino acids were specified by the second codon position, i. e., codons with U in the second position were hydrophobic, whereas most codons with A in the second position were hydrophilic.

The arguments by Woese et al. [75] that amino acids coded by C in the second codon position seem to have similar polar requirement indicate that these similar amino acids were among the first group of amino acids recognized by ancestor tRNAs. The results of our constraint analysis agree with this conclusion and indicate that codons with C in the second position may be the earliest codons to define the modern genetic code.

A striking feature of the timelines of codon discovery of our study was that the most ancient codons belonged to type I tRNA molecules with the most ancient charging functions (Ala and Pro) and that the most ancient charging functions of type II tRNA (Sec, Tyr, Ser, and Leu) had codons that were much more derived. Even when we excluded from constraints type I tRNA take-over molecules coding for Tyr, Ser, and Leu that we identified in our trees (Figure 1), *S* values did not match those of codons for Pro and Ala (with an important exception in the codon for Tyr) (Figure 4). This strongly supports the existence of a fundamental take-over in the evolution of the genetic code, in which ancient type II tRNA molecules had their functional identities replaced relatively late in evolution by a modern code. This modern code may have originated when type II tRNA structures lost their long variable arms and associated identity elements. The ancient functional identity of type II tRNA molecules could have been embedded in the ‘anticodon’ arm as a primitive code that is inexistent today [79] or perhaps more probably in the acceptor arm of the tRNA molecule that is known to harbor an operational code that is older and partially complementary to the classic genetic code [70]–[73], [80]. At present, we cannot establish the actual details of this recruitment event. Regardless of how this take-over took place, results suggest the classic genetic code is quite modern and arose well after amino acid charging functions were in place. Moreover, the lack of correlation between ancestries of amino acid charging and ancestries of codons suggest the discovery of these functions in evolution were for the most part unlinked and the result of different histories of recruitment (Figure 4). This is consistent with evolutionary profiles related to aminoacyl-tRNA synthetases and the emerging phylogenetic picture that suggests these enzymes played a minimal role in the evolution of the genetic code [81].

While tRNA molecular identity appears to have been established in evolution prior to cognate aaRSs [82], [83], we see groups of functions that are clustered in our ancestry plot (Figure 4) and match class I and class II aaRS superclusters previously defined by sequence and structural analyses [84]. These classes of molecules probably developed their functionalities concurrently in evolution. Specifically, functions associated with class I aaRS superclusters GluRS-GlnRS and TrpRS-TyrRS appeared clearly linked in our ancestry plot. Functions of class I supercluster ValRS-IleRS-LeuRS-MetRS were however split into three groups, with functions of IleRS and ValRS clustered together. Those associated with class II aaRS superclusters were also linked in the plot. Functions of supercluster ThrRS-ProRS-SerRS appeared more ancient than those of supercluster LysRS-AspRS-AsnRS, and functions of GlyRS and HisRS formed separate groups, consistent with structural analyses of the proteins [84]. It is remarkable that evolutionary patterns in aaRSs matched those in tRNA molecules, despite the confounding effects of recruitment. This suggests co-evolution between tRNA and cognate synthetases of protein and perhaps ribozyme origin.

### Conclusions

Since it was deciphered [69], the evolution of genetic code has been the subject of much study. However, the expansion of amino acids building blocks through evolution has been generally linked to the evolution of the genetic code. We provide here clear indication that the evolution of these two tRNA functions was unlinked. We focus on how function (amino acid charging and codon assignment identity) evolved in the reconstructed trees derived from sequence and structure of tRNA molecules by using novel phylogenetic methods. Our results revealed the effects of recruitment processes and how these have impacted the history of this molecule. The use of constraint analyses uncovered disjoint evolutionary patterns associated with evolution of amino acid specificity and codon identity, indicating that co-options and take-overs embedded perhaps in horizontal gene transfer affected differentially the amino acid charging and codon identity functions. The proposed timelines of amino acid charging showed for example that type II tRNA molecules were ancient and sustained important take-overs related to codon identity. However, the timelines of codon history showed the importance of the second and then the first codon position in evolution and revealed several appealing patterns, including a role of strength of hydrogen bonds in the birth of the genetic code. Our results appear for the most part consistent with recent statistical analyses of tRNA sequences that support a strand symmetric ancient world in which tRNA had both a genetic and functional role [85].

Phylogenies reconstructed from the structure of several functional RNA molecules at different taxonomical levels (from the subspecies/species levels to the universal tree) generally matched phylogenies reconstructed from sequence (e.g., [22], [25], [26], [86]–[88]). While this supports the validity of the method, it also reveals congruent phylogenetic signals in the sequence and structure of the molecules examined. A number of recent studies have used the sequences of specific tRNA isoacceptors to build trees delimiting the three superkingdoms of life (e.g., tRNA^Lys^ [82], tRNA^Cys^ [83]; tRNA^Asn^ and tRNA^Gln^ [89]). However, tRNA phylogenies that incorporate structural information, as those presented here, generally failed to group tRNAs belonging to individual superkingdoms into monophyletic groups, with the exception of some isoacceptor-specific trees [28]. Since diversification of tRNA structure appears to predate organismal diversification [27], [34], we reason structures carry deep phylogenetic signal while sequences embed more recent molecular history. This explains lack of congruence between phylogenies reconstructed from slow evolving, ancient structures and phylogenies reconstructed from sequences, which change at faster pace. This scenario is supported by the existence of vast networks in sequence space defining common structures that expand when structures evolve for reduced conformational plasticity and increase molecular order [90]. We here show that deep phylogenetic signal in tRNA structure can be nevertheless mined efficiently with the tools of phylogenetic constraint.

## Materials and Methods

### Data

part 2 (compilation of trna sequence) of the bayreuth trna database (http://www.uni-bayreuth.de/departments/biochemie/trna; September 2004 edition) contains a total of 571 tRNA sequences for which there is information about base modifications. These tRNAs have cloverleaf secondary structures that were derived by comparative analysis using an alignment that is most compatible with tRNA phylogenies and known 3-dimensional models of structure [91], [92]. We took the entire data set and scored a total of 42 structural characters describing geometrical features of tRNA molecules, establishing character homology by the relative position of substructures in the cloverleaf [27], [28]. We coded the length (the total number of bases or base pairs) and number of the substructures as character states and defined them in alphanumerical format with numbers from 0 to 9 and letters from A to F. We gave the minimum state (0) to missing substructures. Modified bases were treated as deviations from the cloverleaf model and were not allowed to establish canonical Watson-Crick pairs. We scored each helical stem region as two complementary sequences (5′ and 3′ sides). We partitioned the dataset into subsets categorized by molecules belonging to superkingdoms (Archaea, Bacteria, and Eukarya) or viruses/bacteriophages, charging functions, or codon identity. In this study, we invoked a “total evidence” approach [93], [94] (also called “simultaneous analysis” [95]) in phylogenetic analysis to combine both sequence and structure data of the complete (571 tRNAs) and partitioned matrices. The goal was to provide stronger support for the phylogenetic groupings recovered from analyses of structural data. A total of 99 characters were scored from aligned tRNA sequences.

### Character coding

We treated observable features describing the structure of molecules as phylogenetic multi-state characters. These characters exhibit character states, variants of each structural feature that is homologous. Our characters transform from one character state to another along linearly ordered and reversible pathways in which a particular path of possible evolution is specified. In particular, we treat geometrical features in structure as linearly ordered characters because RNA structures evolve in discrete manner by adding and deleting nucleotide units. This generates gradual extension or contraction of geometrical features, disfavoring the possible but costly insertion or deletion events. We defined the direction of the evolutionary path by polarizing our character transformation series, i.e. by identifying the ancestral (plesiomorphic) and derived (apomorphic) states in the sequence. In order to polarize structural characters we assume the existence of a generalized evolutionary trend in RNA structure that maximizes molecular order. This results in reversible character transformation sequences that are directional and show asymmetry between character gains and losses. We defined the maximum and minimum character states as the ancestral states for structures that stabilize (stems, modified bases, and G:U base pairs) and destabilize tRNAs (bulges, hairpin loops, and other unpaired regions), respectively.

### Character argumentation

The use of ordered and polarized multistate phylogenetic characters that describe the geometry and statistical properties of the structure of RNA molecules has been discussed in detail elsewhere [22], [23], [25]–[28]. Character argumentation is however important because conclusions about molecular origins depend on the axiomatic component of our models that establishes which are the ancestral states. The polarization hypothesis towards order invokes a general tendency of molecules to be more stable, less plastic (more unique), and more modular [*sensu* Ancel and Fontana [90]), and this tendency is falsifiable. So far, a considerable body of theoretical and experimental evidence has supported these polarization trends:

- *Molecular mechanics*. Comparative studies of extant and randomized sequences show that evolution enhances conformational order and diminishes conflicting molecular interactions over those intrinsically acquired by self-organization [25], [96]–[102]. In fact, randomizations of mono- and dinucleotides have been used to dissect the effects of composition and order of nucleotides in the stability of folded nucleic acid molecules and uncover evolutionary processes acting at RNA and DNA levels [103]. In recent bench experiments, extant evolved RNA molecules encoding complex, functional structural folds were compared to oligonucleotides corresponding to randomized counterparts [104]. Unlike evolved molecules, arbitrary sequences were prone to having multiple competing conformations. In contrast to arbitrary proteins, which rarely fold into well-ordered structures [105], these arbitrary RNA sequences were however quite soluble and compact and appeared delimited by physicochemical constraints such as nucleotide composition that were inferred in previous computational studies [106].
- *Thermodynamics.* The use of thermodynamic principles generalized to account for non-equilibrium conditions experimentally have also verified a molecular tendency towards order that could drive biological change [107]. In fact, the impact of thermodynamic principles in living systems (the “building order from disorder” concept championed by Schrödinger [108]) manifests through optimization of more modern thermodynamic quality descriptors of energy gradients (e.g., maximization of exergy) in non-equilibrium systems that are open to flows of energy and matter [109], [110]. This optimization results in more efficient degradation of incoming (solar) energy through autocatalytic, self-assembly, reproduction, evolution and adaptation processes acting on molecular structures, all of which enhance the order of the system to decrease energy gradients and oppose disequilibrium (in line with second law of thermodynamics). This optimization has important consequences for evolution of molecular structure and the mapping of sequence to structure spaces, which represent different levels of biological organization. For example, RNA molecules have low informational entropy in sequence space, but in structure space highly evolvable phenotypes are also more entropic [111], suggesting increases in the order at one level of organization are counteracted by decreases in the order of the next. This ultimately encourages escape (evolvability) from constraints of order (stasis through structural canalization). Note that a large body of theoretical evidence supports these sequence-to-structure mappings and their consequences on the energetic and kinetic landscape of the evolving molecules [90.112,113], with some important predictions confirmed experimentally in *in vitro* evolution of ribozymes [114].
- *Phylogenetics.* Finally and more importantly, tendencies towards structural order and the rooting of trees have been experimentally supported by phylogenetic congruence of trees reconstructed using geometrical and statistical structural characters [25], [26], [28] and from sequence, structure, and genomic rearrangements at different taxonomical levels, which also match statements from traditional organism classification [22], [23], [25], [26], [86]–[88]. For example, the same phylogenies are produced when using characters that describe the topology of tRNA or characters that describe a molecular morphospace [100] defined by the Shannon entropy of the base-pairing probability matrix, base-pairing propensity, and mean length of helical stem structures of tRNA molecules [28]. Remarkably, polarizing characters in the opposite direction generated trees that were less parsimonious and had topologies incompatible with taxonomical knowledge. Other more indirect results derived from using our focus on structure proved to be congruent, such as hypotheses of organismal origin that used global trees of tRNA structures and constraint analysis [27] and phylogenies of proteomes derived from an analysis of protein structure in entire genomic complements [33]. Every new instance of congruence provides important support to our hypothesis of polarization.

### Phylogenetic analysis

We used maximum parsimony (MP) to search for the most parsimonious trees, i.e., solutions that require the least amount of change. We analyzed all data matrices using equally weighted MP as the optimality criterion in PAUP* v. 4.0 [115]. Our selection of MP over maximum likelihood (ML) approaches is particularly suitable. For example, in our analyses we decrease the likelihood of revisiting the same character state on the underlying tree by using multi-state characters and provide conditions for characters to evolve with equal probability but varying rates, making ML precisely MP [116], [117]. MP trees were reconstructed using heuristic search strategies. Specifically, 1,000 heuristic searches were initiated using random addition starting taxa, with tree bisection reconnection (TBR) branch swapping and the MulTrees option selected. One shortest tree was saved from each search. We included the hypothetical ancestors in the searches for the most parsimonious trees using the Ancstates command. For all the phylogenetic trees, we calculated the bootstrap support (BS) values [118] from 10^5^ replicate analyses using “fast” stepwise addition of taxa in PAUP*. We also calculated the g_1_ statistic of skewed tree length distribution from 10^4^ random parsimony trees to assess the amount of nonrandom structure in the data [119].

### Constraint analysis

Constraint analysis generally restricts the search of optimal trees to pre-specified tree topologies delimiting specific monophyletic groups. Here we used constraint analyses to explore alternative or compare non-mutually exclusive hypotheses of tRNA groupings. The number of additional steps (*S*) required to force particular taxa into a monophyletic group was obtained by using the enforce topological constraint option of PAUP*. The values of *S* circumscribe an evolutionary distance that can be used to quantitatively contrast alternative phylogenetic hypotheses or to compare hypotheses that are not mutually exclusive. We used the latter approach to construct evolutionary timelines. This method was used previously to establish the evolutionary timeline of organismal diversification [27]. In the present study, we conducted constraint analyses on the basis of amino acid specificity (including the ancestry of groups of amino acids circumscribed by various authors), the first, second, third, or the first two bases of the codons (i.e., the third, second, first, or the last two bases of the anticodon).

## Supporting Information

Table S1 (0.06 MB PDF)

Figure S1 The global phylogenetic tree of tRNA molecules with labeled terminal taxa. This tree is shown in four parts due to its size. For every tRNA, species name is followed by the anticodon (symbols of modified bases are adopted from the BAYREUTH tRNA DATABASE), amino acid specificity, and if any, a number to indicate the presence of multiple accessions. tRNAs derived from viruses are indicated with V. Numbers above the branches are bootstrap values. tRNAs with long variable arms are highlighted in pink, while those specifying for Tyr, Leu, and Ser with short variable arms are highlighted in red. Symbols used to describe modified bases in anticodon sequences: ., unknown nucleotide; H, unknown modified adenosine; [, 2-methylthio-N6-threonylcarbamoyladenosine; I, inosine; <, unknown modified cytidine; B, 2′-O-methylcytidine; M, N4-acetylcytidine; }, lysidine; >, 5-formylcytidin; °, 2-O-methyl-5-formylcytidin; ;, unknown modified guanosine; K, 1-methylguanosine; #, 2′-O-methylguanosine; 7, 7-methylguanosine; Q, queuosine; 8, mannosyl-queuosine; 9, galactosyl-queuosine; N, unknown modified uridine; {, 5-methylaminomethyluridine; 2, 2-thiouridine; J, 2′-O-methyluridine; &, 5-carbamoylmethyluridine; 1, 5-methoxycarbonylmethyluridine; S, 5-methylaminomethyl-2-thiouridine; 3, 5-methoxycarbonylmethyl-2-thiouridine; V, uridine 5-oxyacetic acid; 5, 5-methoxyuridine; !, 5-carboxymethylaminomethyluridine; $, 5-carboxymethylaminomethyl-2-thiouridine; ), 5-carboxymethylaminomethyl-2′-O-methyluridine; P, pseudouridine; ], 1-methylpseudouridine.
(1.16 MB PDF)

Figure S2 The global phylogenetic tree of tRNA molecules with labeled terminal taxa described as a phylogram. This tree is shown in four parts due to its size. tRNAs are labeled as described in Figure S1.
(1.06 MB PDF)

Figure S3 Phylogenetic trees of tRNAs derived from maximum parsimony analyses of 17 partitioned data matrices. A. Bacillus subtilis. B. Bos Taurus. C. Drosophila melanogaster, D. Escherichia coli. E. Halobacterium cutirubrum. F. Haloferax volcanii. G. Homo sapiens. H. Lupinus spp. I. Mus musculus. J. Mycoplasma capricolum. K. Neurospora crassa. L. Nicotiana spp. M. Phage. N. Phaseolus vulgaris. O. Saccharomyces cerevisiae. P. Rattus norvegicus. Q. Spinacia oleracea. Terminal leaves are labeled as anticodons (symbols of modified bases are defined in Figure S1) followed by amino acid specificities and if any, a number to indicate the presence of multiple accessions. Numbers above the branches are bootstrap values. Type II tRNA molecules are highlighted in red. Detailed descriptions of the trees are given in Table S1.
(0.46 MB PDF)
