# Supplementary material for: Evolutionary Patterns in the Sequence and Structure of Transfer RNA: A Window into Early Translation and the Genetic Code
Source: PLoS One. 2008 Jul 30;3(7):e2799. doi: 10.1371/journal.pone.0002799 (PMC2474678; doi:10.1371/journal.pone.0002799)
Supplement: Table S1 — (0.06 MB PDF) [file pone.0002799.s001.pdf]

Table S1. Statistics of phylogenetically reconstructed trees of tRNA structures derived from 17 data matrices. Number of taxa analyzed is shown in parentheses. CI = consistency index (with/without uninformative characters), RI = retention index, RC = rescaled consistency index.

**Taxa**	**No. of trees**	**Tree length**	**CI**	**RI**	**RC**	**g_1**
Bacillus subtilis (19)	1	131	0.626/0.566	0.739	0.463	–0.691
Bos taurus (33)	23	355	0.369	0.656	0.242	–0.545
Drosophila melanogaster (13)	3	166	0.633/0.585	0.653	0.413	–0.827
Escherichia coli (45)	502	216	0.454/0.413	0.761	0.345	–0.589
Halobacterium cutirubrum (12)	2	116	0.672/0.604	0.568	0.382	–0.537
Haloferax volcanii (41)	40	209	0.450/0.419	0.739	0.332	–0.467
Homo sapiens (22)	1	287	0.453/0.416	0.588	0.266	–0.422
Lupinus spp. (13)	5	161	0.615/0.566	0.523	0.322	–0.450
Mus musculus (17)	9	186	0.618/0.580	0.689	0.426	–0.731
Mycoplasma capricolum (29)	12	168	0.548/0.500	0.675	0.370	–0.704
Neurospora crassa (10)	2	158	0.658/0.609	0.518	0.341	–0.728
Nicotiana (11)	2	126	0.659/0.606	0.682	0.449	–0.309
Phage (13)	2	145	0.662/0.602	0.608	0.403	–0.828
Phaseolus vulgaris (21)	74	170	0.606/0.573	0.796	0.483	–0.411
Rattus norvegicus (27)	10	229	0.480/0.466	0.749	0.36	–0.617
Saccharomyces cerevisiae (51)	1521	383	0.339/0.336	0.689	0.234	–0.288
Spinacia oleracea (11)	6	129	0.682/0.637	0.568	0.388	–0.722
