# Supplementary figures and images for: Evolutionary Patterns in the Sequence and Structure of Transfer RNA: A Window into Early Translation and the Genetic Code
Source: PLoS One. 2008 Jul 30;3(7):e2799. doi: 10.1371/journal.pone.0002799 (PMC2474678; doi:10.1371/journal.pone.0002799)

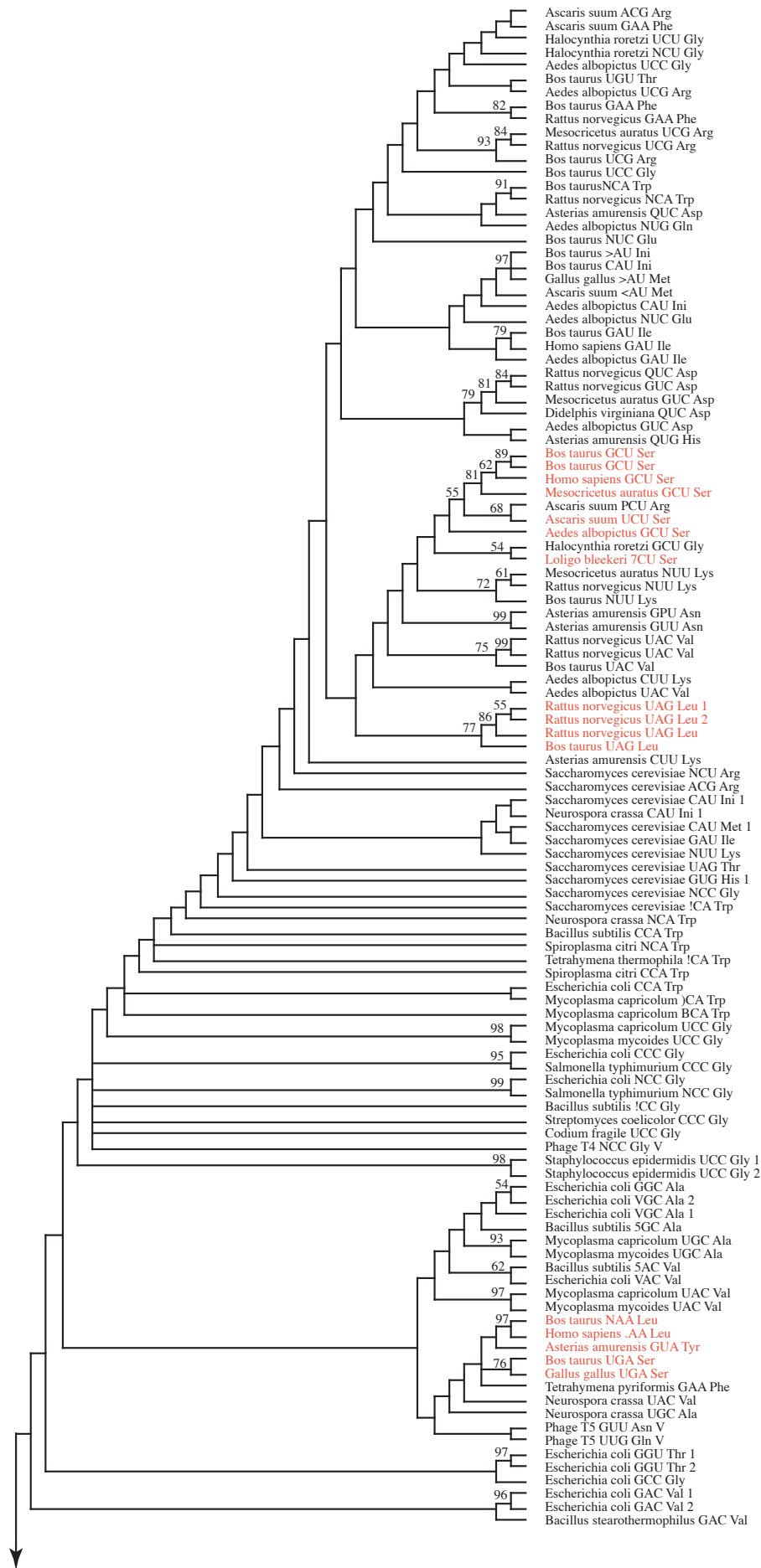

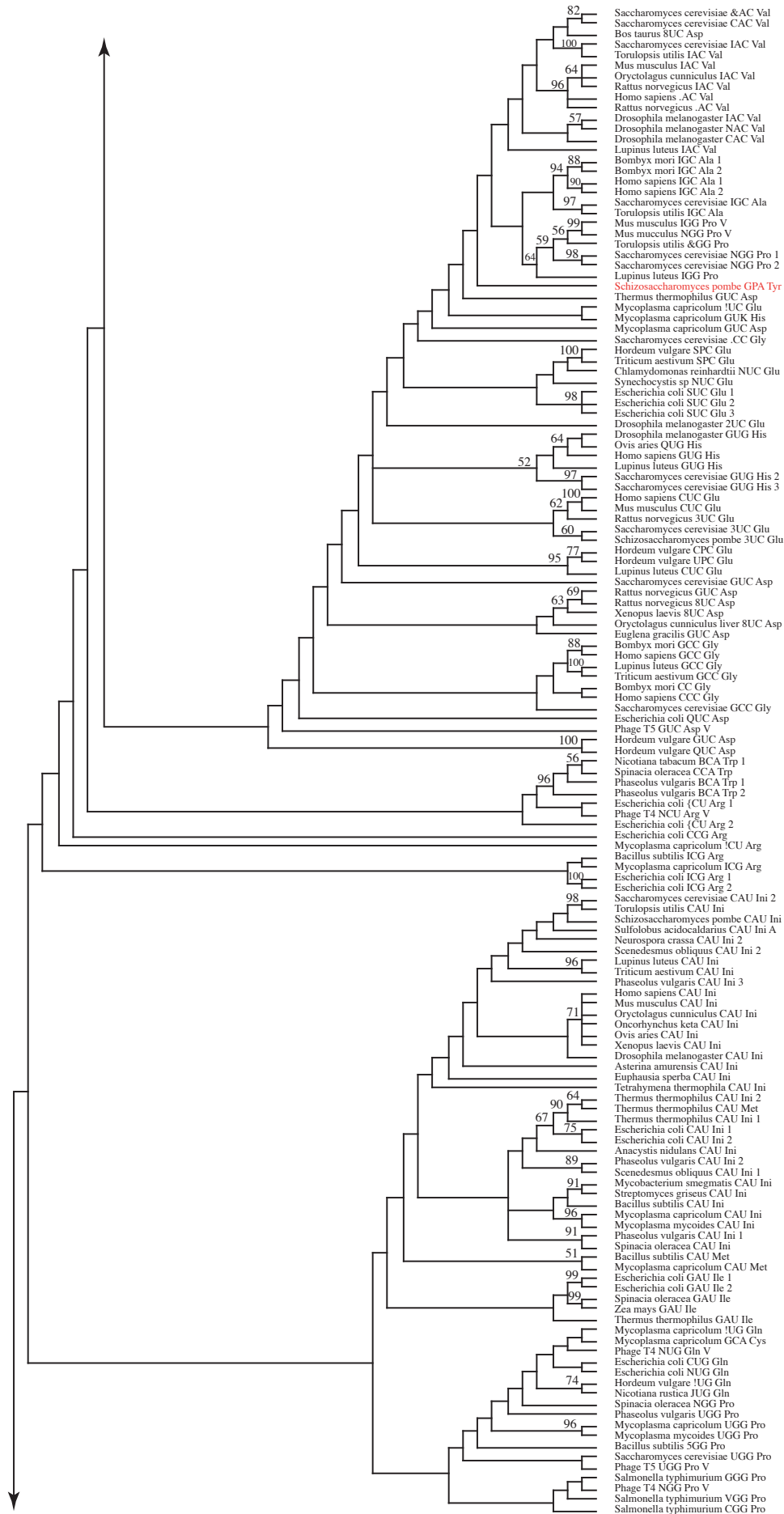

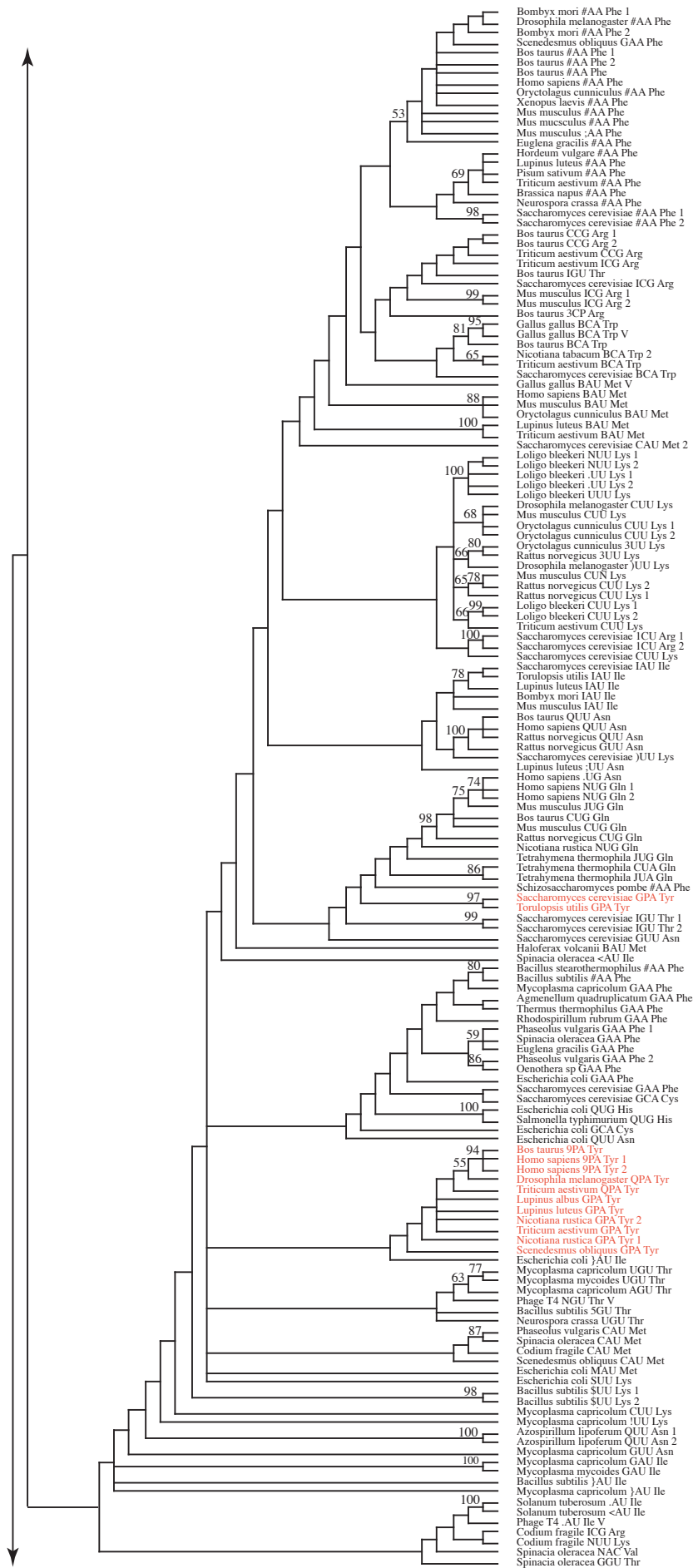

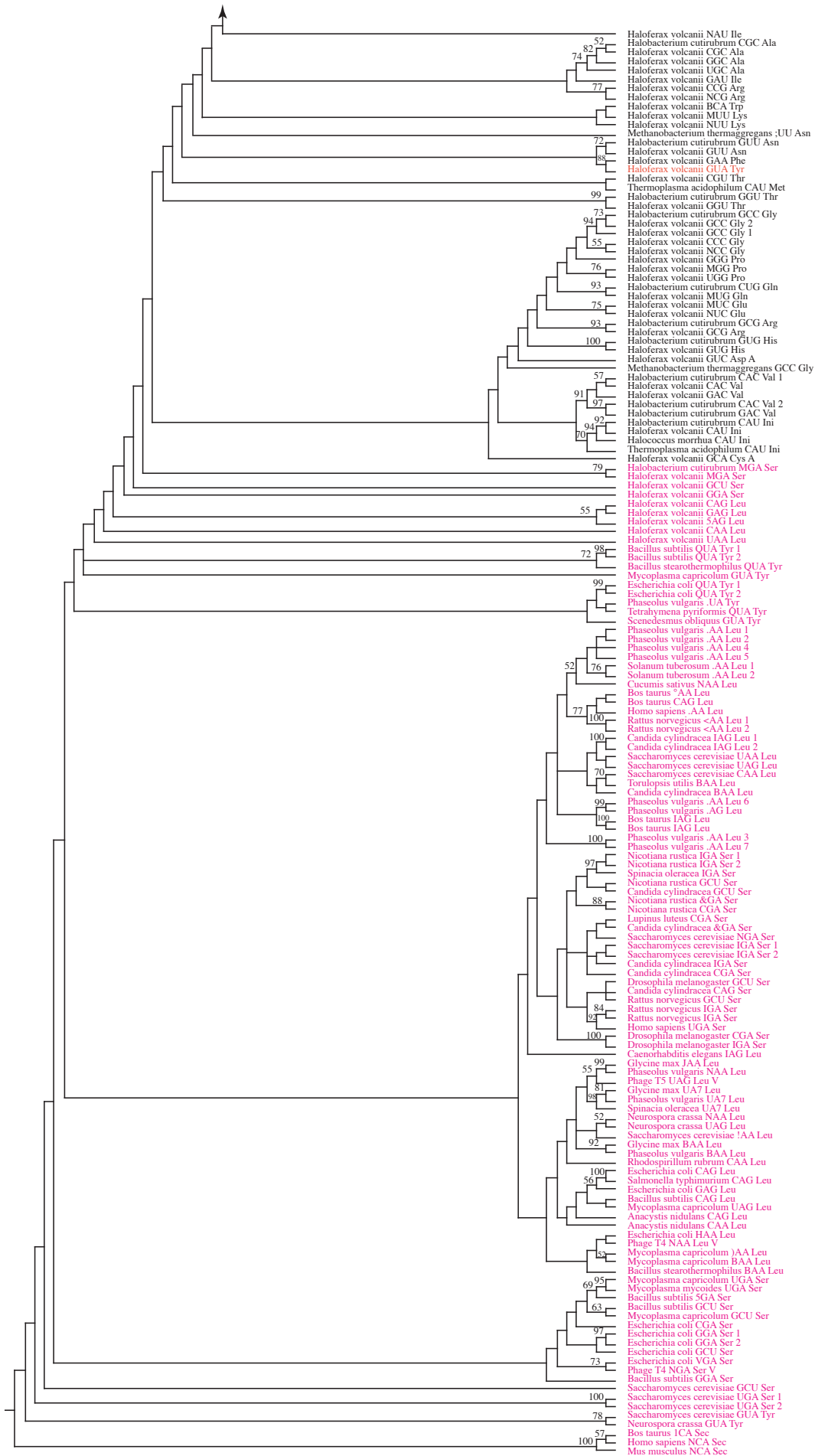

Supplement: Figure S1 — The global phylogenetic tree of tRNA molecules with labeled terminal taxa. This tree is shown in four parts due to its size. For every tRNA, species name is followed by the anticodon (symbols of modified bases are adopted from the BAYREUTH tRNA DATABASE), amino acid specificity, and if any, a number to indicate the presence of multiple accessions. tRNAs derived from viruses are indicated with V. Numbers above the branches are bootstrap values. tRNAs with long variable arms are highlighted in pink, while those specifying for Tyr, Leu, and Ser with short variable arms are highlighted in red. Symbols used to describe modified bases in anticodon sequences: ., unknown nucleotide; H, unknown modified adenosine; [, 2-methylthio-N6-threonylcarbamoyladenosine; I, inosine; <, unknown modified cytidine; B, 2′-O-methylcytidine; M, N4-acetylcytidine; }, lysidine; >, 5-formylcytidin; °, 2-O-methyl-5-formylcytidin; ;, unknown modified guanosine; K, 1-methylguanosine; #, 2′-O-methylguanosine; 7, 7-methylguanosine; Q, queuosine; 8, mannosyl-queuosine; 9, galactosyl-queuosine; N, unknown modified uridine; {, 5-methylaminomethyluridine; 2, 2-thiouridine; J, 2′-O-methyluridine; &, 5-carbamoylmethyluridine; 1, 5-methoxycarbonylmethyluridine; S, 5-methylaminomethyl-2-thiouridine; 3, 5-methoxycarbonylmethyl-2-thiouridine; V, uridine 5-oxyacetic acid; 5, 5-methoxyuridine; !, 5-carboxymethylaminomethyluridine; $, 5-carboxymethylaminomethyl-2-thiouridine; ), 5-carboxymethylaminomethyl-2′-O-methyluridine; P, pseudouridine; ], 1-methylpseudouridine. (1.16 MB PDF) [file pone.0002799.s002.pdf]

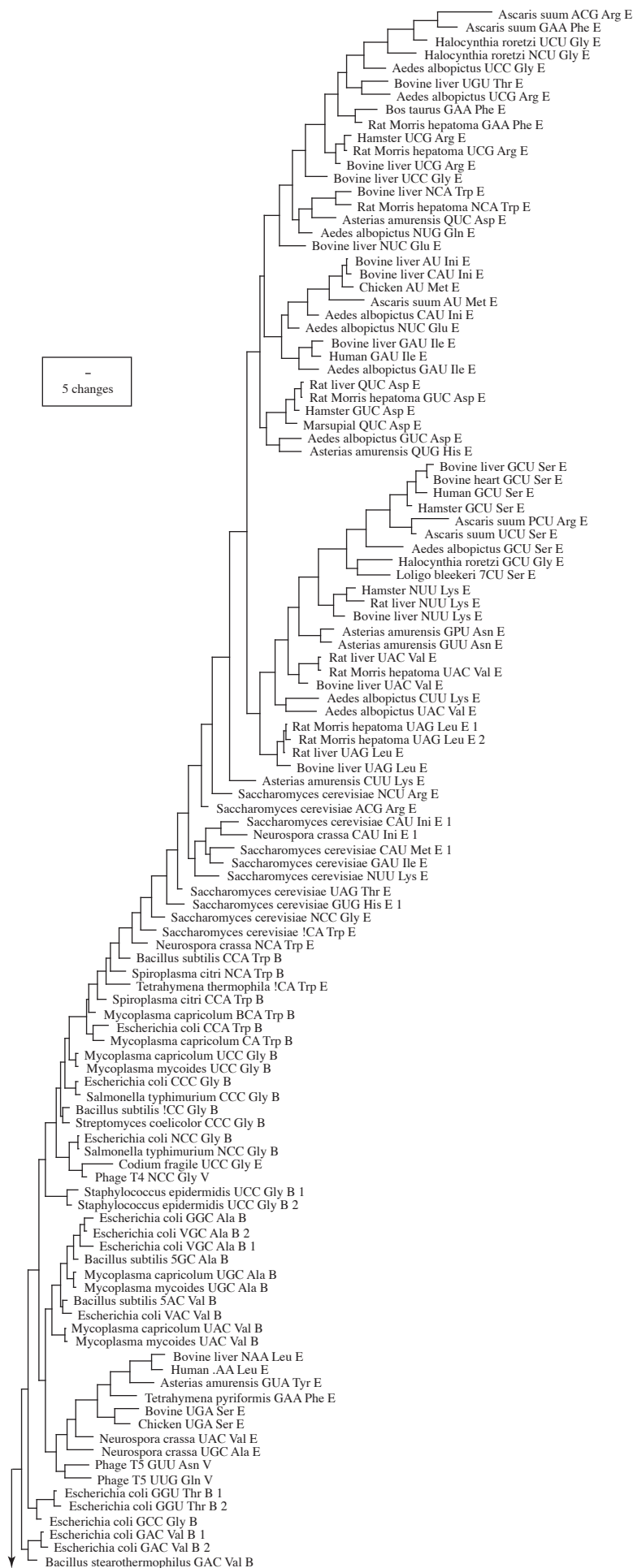

5 changes

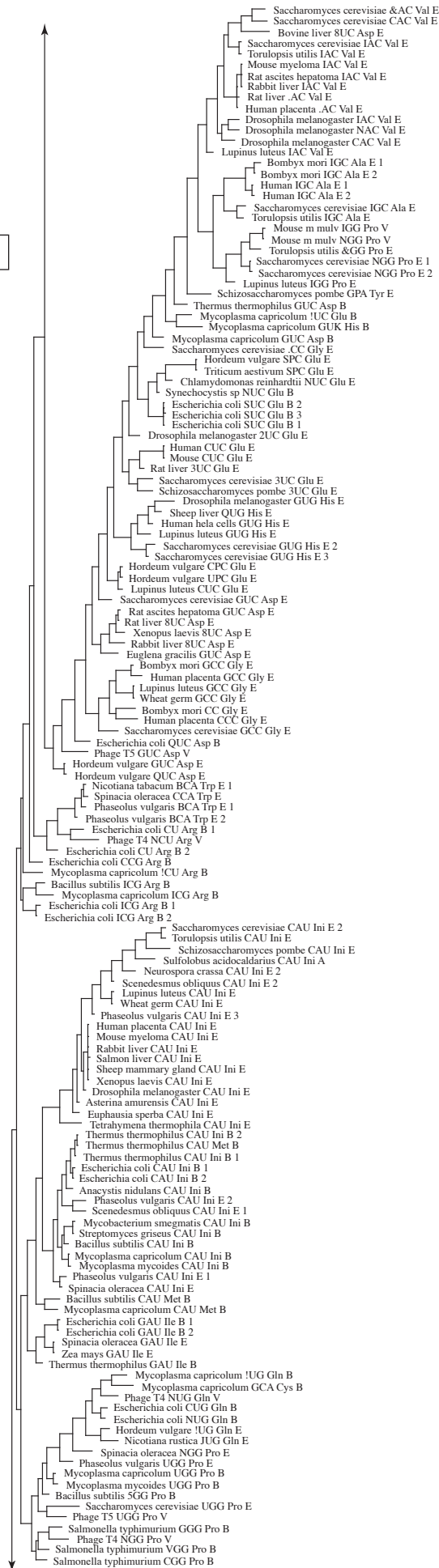

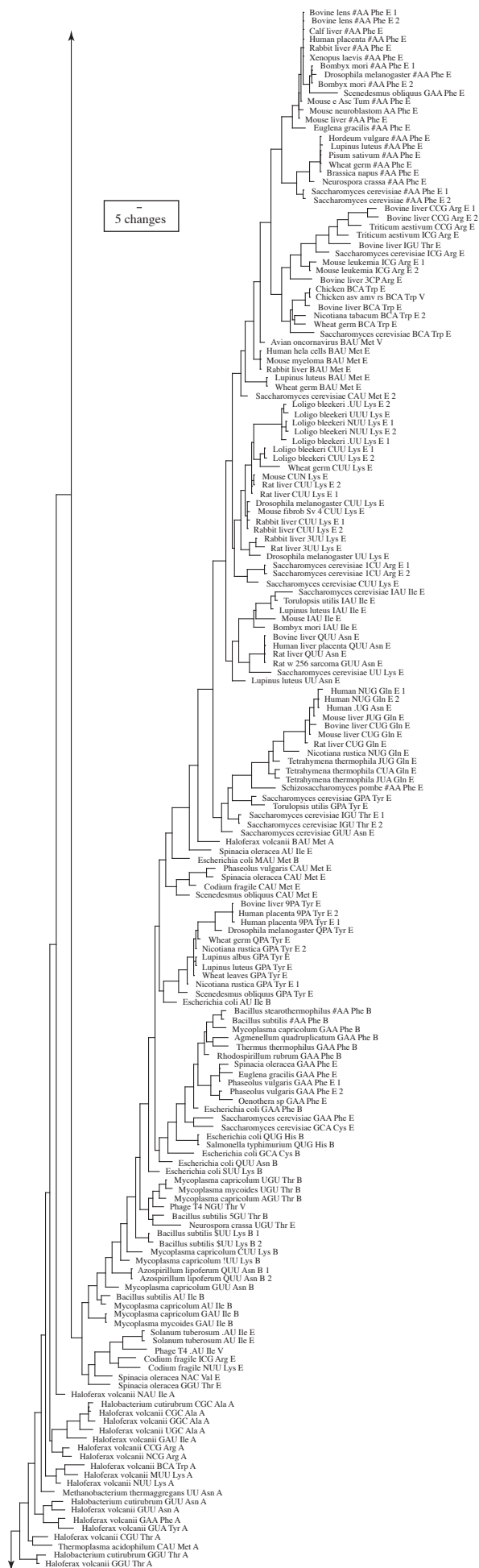

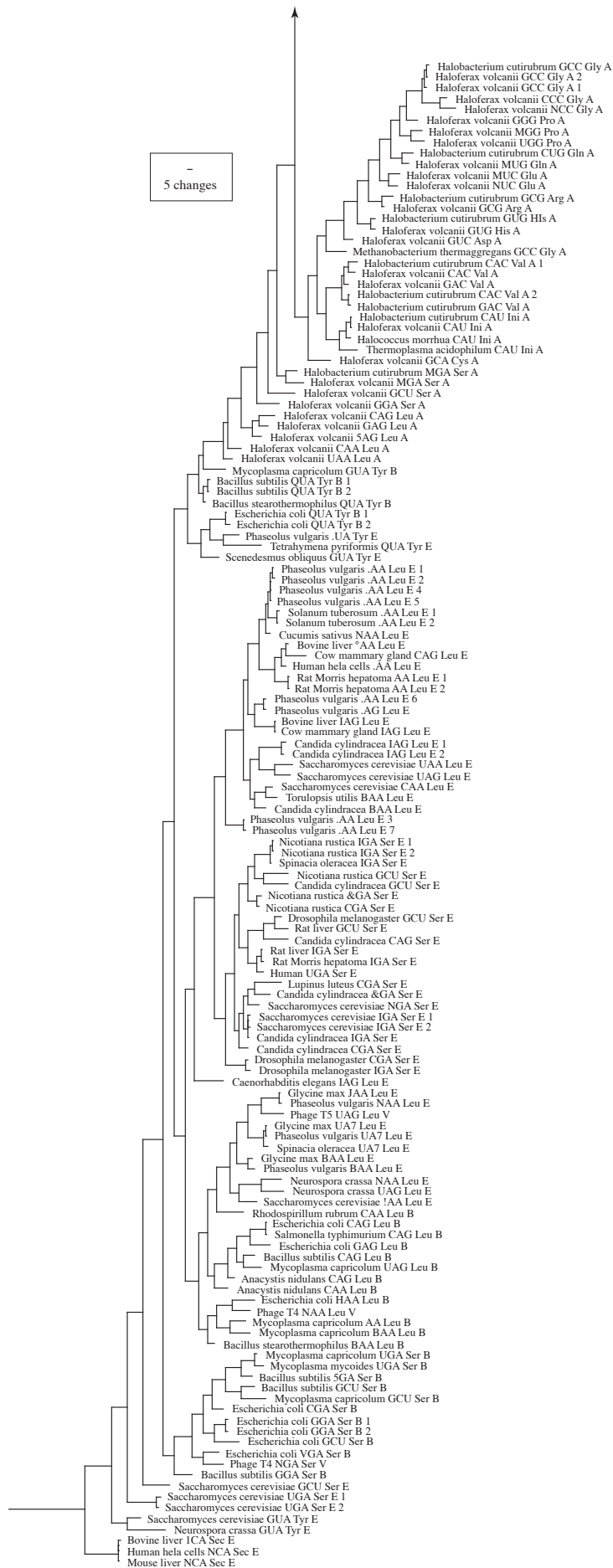

Supplement: Figure S2 — The global phylogenetic tree of tRNA molecules with labeled terminal taxa described as a phylogram. This tree is shown in four parts due to its size. tRNAs are labeled as described in Figure S1. (1.06 MB PDF) [file pone.0002799.s003.pdf]

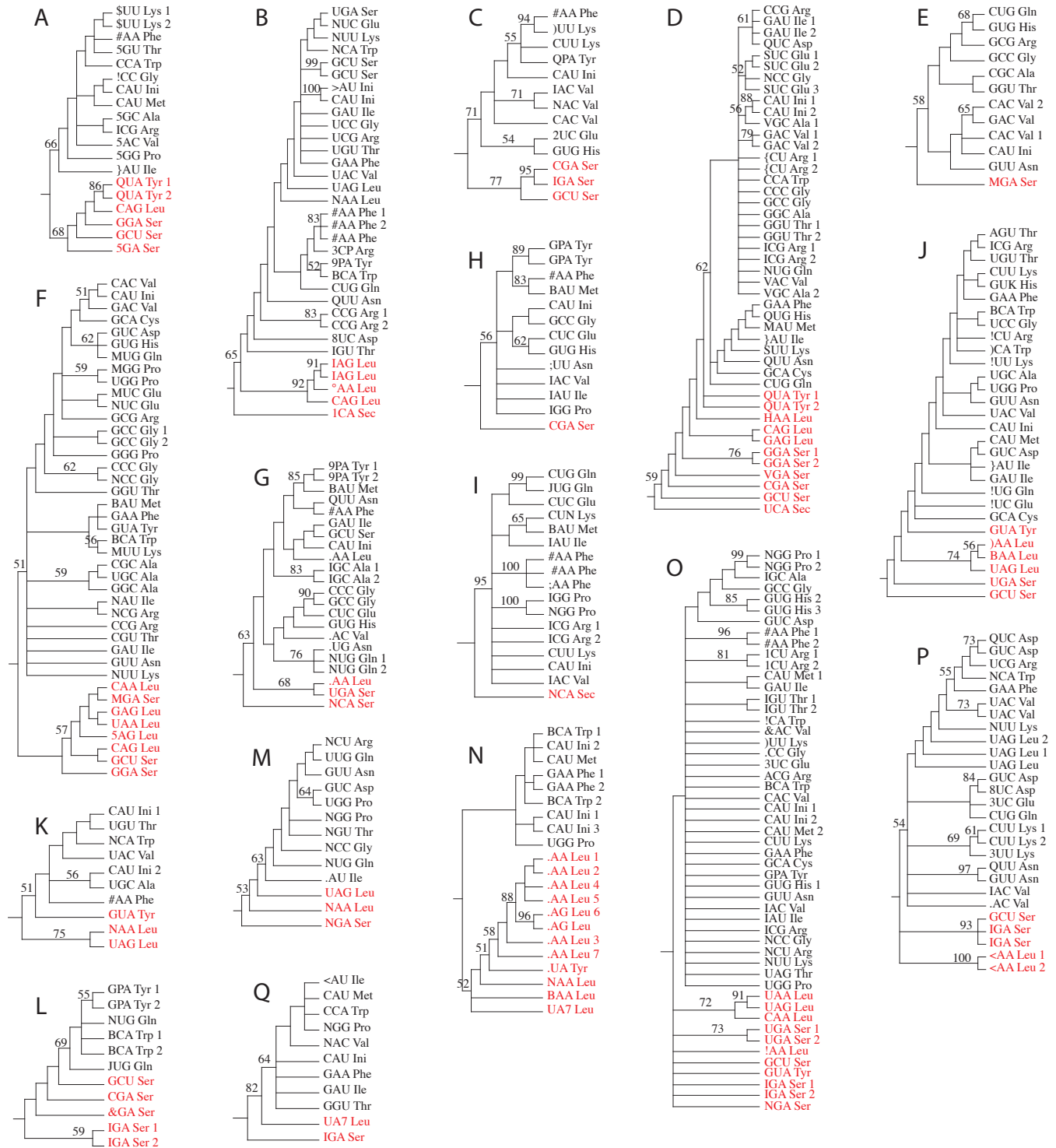

Supplement: Figure S3 — Phylogenetic trees of tRNAs derived from maximum parsimony analyses of 17 partitioned data matrices. A. Bacillus subtilis. B. Bos Taurus. C. Drosophila melanogaster, D. Escherichia coli. E. Halobacterium cutirubrum. F. Haloferax volcanii. G. Homo sapiens. H. Lupinus spp. I. Mus musculus. J. Mycoplasma capricolum. K. Neurospora crassa. L. Nicotiana spp. M. Phage. N. Phaseolus vulgaris. O. Saccharomyces cerevisiae. P. Rattus norvegicus. Q. Spinacia oleracea. Terminal leaves are labeled as anticodons (symbols of modified bases are defined in Figure S1) followed by amino acid specificities and if any, a number to indicate the presence of multiple accessions. Numbers above the branches are bootstrap values. Type II tRNA molecules are highlighted in red. Detailed descriptions of the trees are given in Table S1. (0.46 MB PDF) [file pone.0002799.s004.pdf]
